# PDA Stenting May Be a Favorable Alternative to BTT Shunt Regarding Acute Complications and Mid Term Pulmonary Artery Growth

**DOI:** 10.1007/s00246-025-03845-1

**Published:** 2025-04-11

**Authors:** Paz Rimoni, Ori Stern, Gur Mainzer, Uriel Katz, Amir Vardi, David Mishali, Sharon Borik Chiger

**Affiliations:** 1 Faculty of Medicine, Tel Aviv University, Tel Aviv, Israel; 2 Pediatric Heart Institute, Edmond and Lily Safra Children’s Hospital, Sheba Medical Center, Tel Hashomer, Israel

**Keywords:** Blalock–Thomas–Taussig shunt, PDA stenting, CHD, Pulmonary artery stenosis

## Abstract

The Blalock Taussig Thomas Shunt (BTTS) is a palliative procedure for children with congenital heart disease (CHD). This operation may involve significant morbidity and mortality, and its effect on pulmonary arteries has been ambivalent. Ductal stenting is an alternative option. We aimed to evaluate pulmonary artery growth and clinical course following an operative BTTS as compared to percutaneous ductal stenting. Retrospective review of 92 patients who underwent BTTS and 34 patients who underwent percutaneous ductal stenting at the Sheba Medical Center between the years 2014–2021. Clinical data were extracted from patient medical files for assessment of morbidity, mortality, and procedural complications. Echocardiograms and angiography preformed before, six months and two years following the procedure were evaluated for pulmonary artery assessment. The groups did not differ significantly in terms of demographic findings, degree of complicated cases, or presence of antegrade pulmonary blood flow before the procedure. PDA stent patients had shorter ICU length of stay (median 6 days (IQR 3–8) vs. 9 (7–17); *p* < 0.001), hospitalization (7 days (3–9) vs. 11 (7–18); *p* < 0.001), and ventilation (0.25 days (0.25–2) vs. 3 (1–5); *p* < 0.001). The stent group needed minimal to no ionotropic support on the days 0, 1, and 2 after intervention (vasoactive-inotropic score (VIS) on the day of the procedure 0 (0–0.5) in the stent group vs. 8.7 (2.5–14.2) in the shunt group, VIS on day one 0 (0–2.5) vs. 7.7 (2.5–14.2), VIS on postoperative day two 0 (0–2.1) vs. 5 (0–11); *p* < 0.001). Pulmonary artery stenosis was present in 58.8% of PDA stents before intervention vs. 33.7% of BTTS; *p* = 0.01), yet the PDA stent group showed improved pulmonary artery growth with larger pulmonary artery diameter at both 6 months (McGoon ratio 1.9 (1.7–2.2) vs. 1.7 (1.4–1.9); *p* = 0.001) and 2 years after intervention (McGoon ratio 2.2 ± 0.5 vs. 1.8 ± 0.4; *p* = 0.004). Mortality in the ICU was significantly higher in the BTTS group 13 (14.1%) versus 1 (2.9%) in the PDA stent group (*p* < 0.001), and even higher if BTTS was performed with cardiopulmonary bypass 6 (19.4%) vs PDA stenting 1 (2.9%) (*p* < 0.05). Pulmonary artery assessment showed that late surgical pulmonary artery repair after a PDA stent may be preferable to arterioplasty performed early with an initial BTTS (0% vs. 100% qualitative stenosis, *p* = 0.005). PDA stenting results in significantly fewer complications and lower mortality than BTTS, even if surgery is performed with cardiopulmonary bypass. In addition, PDA stenting allows good and in some cases more favorable mid-term pulmonary artery growth, particularly for patients whose anatomy necessitates surgical pulmonary arterioplasty. PDA stenting may therefore be a better option than BTTS for palliation in duct dependent pulmonary circulation, even for infants with pulmonary artery stenosis, whose initial palliation is a PDA stent followed by pulmonary arterioplasty later in infancy.

**Supplementary Information:**

The online version contains supplementary material available at 10.1007/s00246-025-03845-1.

## Introduction

Infants with cyanotic congenital heart disease with duct dependent pulmonary circulation require early neonatal intervention. Historically, BTTS was not considered a complex procedure, however, adverse outcomes and complications are often reported, with hospital mortality between 13 and 18% depending on the underlying congenital heart defect diagnosis [1]. There are frequent shunt-related complications such as acute thrombosis, shunt stenosis, over shunting and pulmonary artery stenosis [1, 2]. In addition, BTTS was found to cause an increase in the pulmonary arterial index (PAI, also known as Nakata index, a measure of pulmonary artery cross-sectional area relative to body surface area) but also in pulmonary arterial distortion [3, 4].

Since surgical shunt involves significant morbidity and mortality, ductal stenting (DS) has become an alternative palliative strategy to maintain adequate pulmonary blood flow in the neonate with duct dependent pulmonary blood flow [5]. The technique of ductal stenting depends on the underlying cardiac malformation and the morphology of the PDA, and rare complications may occur during the procedure such as acute thrombosis (2–3%) and spasm of PDA (< 1%) [6]. However, when comparing PDA stenting to BTTS surgery, PDA stenting may have several advantages such as shorter hospitalization in the intensive care unit (ICU) and less procedural complications. It may also confer more favorable results on pulmonary artery size and symmetry, although further research is needed to identify patient characteristics who would benefit the most from the PDA stenting procedure [7, 8]. What is more, there is still insufficient information regarding the best timing for surgical correction of the pulmonary arteries in children whose defect includes pulmonary artery stenosis: during early shunt surgery or at a later stage following neonatal ductal stenting.

Our objective was to examine the mid-term outcomes of PDA stenting versus BTTS regarding clinical outcomes and pulmonary artery (PA) growth.

## Methods

We present a historical cohort, single-center study of children who underwent BTTS or PDA stenting at Safra Children's Hospital, Chaim Sheba Tel-Hashomer Medical Center between the years 2014–2021.

Data collection was stopped in 2021. From 2021 to the present day, every patient with duct dependent pulmonary circulation in our medical center has been referred for PDA stenting, with BTTS surgery now being rarely performed.

Our study population included children under the age of 7 months who were diagnosed with congenital defects involving duct dependent pulmonary circulation, both uni- and biventricular and excluded children with hypoplastic left heart syndrome. Children diagnosed with Tetralogy of Fallot (TOF) or Transposition of the Great Arteries (TGA) with biventricular physiology required palliation due to severe pulmonary stenosis or pulmonary atresia. Whereas children with TGA as part of a complex single ventricle defect, were categorized as single ventricle defect. Pulmonary atresia-intact ventricular septum (PA/IVS) were included in this diagnosis group if the child presented with pulmonary atresia and no ventricular septal defect or with critical pulmonary stenosis and duct dependent pulmonary circulation. This group was further categorized as uni- or biventricular physiology according to palliation or treatment pathway, according to tricuspid valve z score, right ventricular size and presence of RV-dependent coronary circulation. All available medical records were reviewed, including follow up data through November 2022.

In the early years, only patients with high risk for surgery were referred for ductal stenting, and there was a preference for ductal stenting in patients with dual supply of pulmonary blood flow. Once the technique was well established, most babies were referred for initial ductal stenting unless significant pulmonary artery stenosis was found. This retrospective cohort includes all patients for whom BTTS or PDA stenting was the initial palliation.

A patient was defined as a complicated case if any of the following were present: known genetic syndrome, prematurity, heterotaxy, and/or late presentation > 30 days. Some children had a phenotypic suspicion of genetic syndrome without clear genotypic diagnosis; those were included under “suspicion of genetic syndrome”. The children who had clear diagnosis were included in the “complicated cases” as having the diagnosis of the genetic syndrome.

Data regarding patient demographics and preoperative clinical condition was reviewed.

The definitive surgery was designated biventricular repair or univentricular palliation, usually a Glenn procedure, in which case the patient was categorized as univentricular physiology, as appropriate. Antegrade flow designates a connection to the pulmonary arteries other than the shunt or stent; that is two sources of pulmonary blood flow. Most of the patients who had previous intervention or surgery were children with PA/IVS or critical pulmonary stenosis. Usually, these patients were initially treated with pulmonary valve perforation as necessary and balloon dilation percutaneously, then taken for another catheterization for PDA stenting if they remained cyanotic.

Clinical outcomes during hospitalization were assessed, such as procedural and post-procedural complications, ICU length of stay, ventilation days, and hospitalization days. Need for inotropic support after the procedure was calculated using the Vasoactive Inotropic score (VIS) [VIS = Dopamine dose (mcg/kg/min) + Dobutamine dose (mcg/kg/min) + 100 × epinephrine dose (mcg/kg/min) + 10 × Milrinone (mcg/kg/min) + 10,000 × Vasopressin dose (units/kg/min) + 100 × Norepinephrine dose (mcg/kg/min)] ) [9]. Calculations were done at four time intervals: on the day of the procedure upon return to the intensive care unit, and 1, 2, and 7 days after. In addition, mortality in ICU or hospital wards after procedure was assessed. Patient survival was assessed 30 days after procedure and overall survival was assessed until the last follow-up. Mid-term outcomes such as need for pulmonary artery (PA) plasty at second intervention and need for reintervention 6 months and 2 years after procedure were assessed.

For patients who received a PDA stent, the sizing protocol was as follows: those weighing over 3 kg were fitted with a 4 mm diameter stent, while those under 3 kg were provided with a 3.5 mm diameter stent. In contrast, for patients undergoing BTTS, a fixed shunt size of 4 mm was utilized regardless of their weight. The shunt side was determined by the arch sidedness; right aortic arch patients received a left BTTS and left aortic arch patients received right BTTS regardless of the side where stenosis was present.

**Fig. 1.**
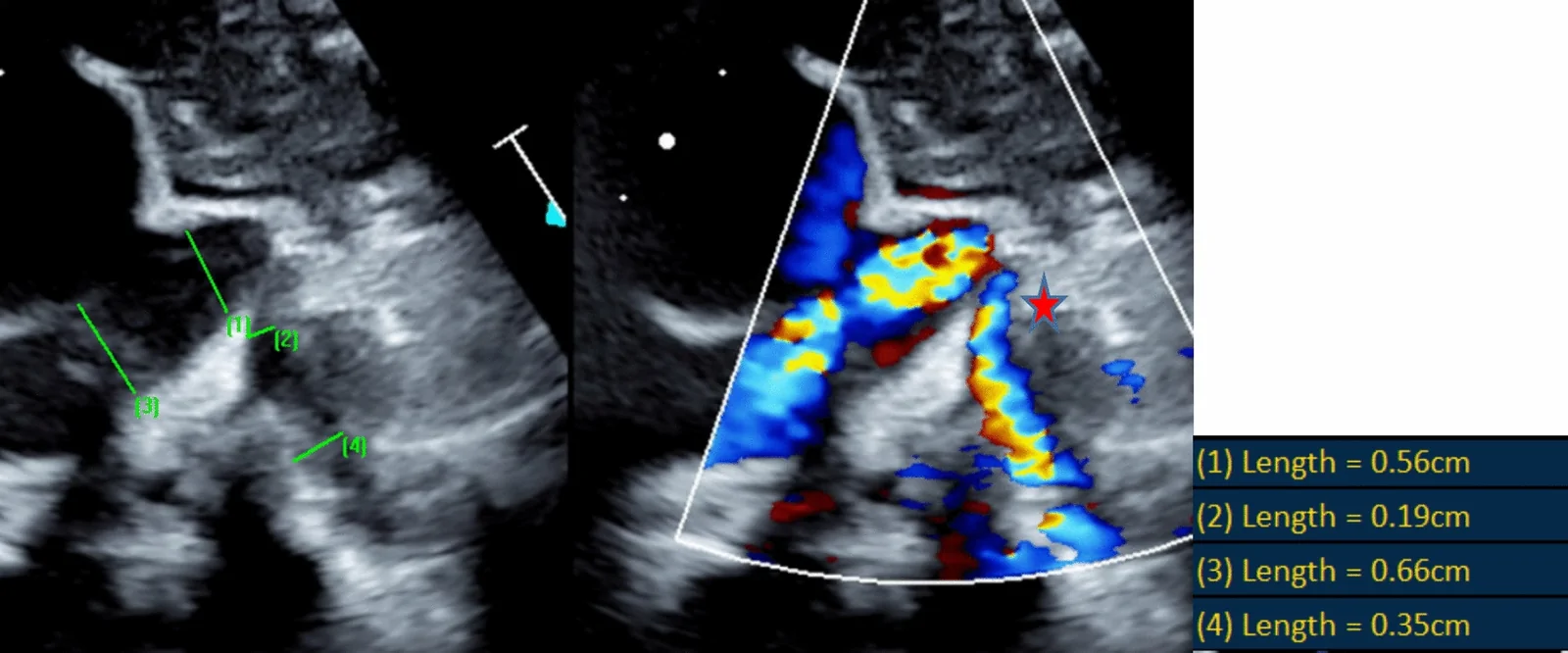
2D vs color Doppler echo images used to assess both categorical pulmonary artery stenosis (star) and quantitative pulmonary artery measurements six months after PDA stenting, (1&2) represent proximal branch pulmonary artery measurements, while (3&4) represent distal measurements used to calculate McGoon ratio on echo

Pulmonary artery size was assessed by serial echocardiograms and angiography. Since many of the patients of the cohort were from outside Israel, serial imaging was not always available, so data regarding pulmonary artery growth were collected retrospectively at set time-points after shunting or stenting, by both echocardiography and angiography. Measurements were done before the procedure, 6 months, and 2 years after the procedure. Categorical pulmonary artery stenosis was indicated if more than 50% reduction in of the PA diameter was present. Quantitative PA assessment included diameter, McGoon Ratio (= RPA diameter + LPA diameter)/Descending aorta diameter)) and Nakata index = (= *π* (1/2*RPA diameter + 1/2*LPA diameter)^2^/Body surface area)) (mm^2^/m^2^) (Fig. 1).

### Statistical Analysis

Categorical variables were described as frequency and percentage. Continuous variables were evaluated for normal distributions using Kolmogorov–Smirnov Test. Continuous variables that were normally distributed were reported as mean and standard deviation, while skewed variables were reported as median and interquartile range. Parameters were compared between the two procedure types. Continuous variables were compared using independent samples T-test or Mann–Whitney test and categorical variables were compared using Chi-square test or with Fisher's exact test. Kaplan–Meier curve and log-rank test were applied to compare overall survival between the two procedures.

All data were summarized in an Excel file, with all statistical tests two sided. For all statistical analysis, we used SPSS version 28, and a *p* value smaller than 0.05 was considered statistically significant. Adjustment of possible confounders was done by matching analysis.

## Results

### Patient Demographics, Clinical and Anatomic Factors

The study included a total of 126 patients. Most patient characteristics did not differ significantly between the ductal stenting and BTTS groups, including percentage of complicated cases or the presence of antegrade pulmonary blood flow (Table 1). In the BTTS group, 55(59.9%) were defined as complicated cases, as compared to 14(41.2%, *p* = 0.063) in the PDA stent group. The median age at intervention was 15 days in the BTTS group and 12 days in the PDA stent group. Out of 126 patients included in the study, only 19 patients were above 30 days of age at intervention. Specifically, 6 patients were 1.5 months old, 2 were 2.5 months old, 6 were 3 months old, 1 patient was 4 months old, 1 was 6 months old and 3 patients were 7 months old. Mechanical ventilation before the intervention was more common among the BTTS group, 44 (47.7%) compared to 8 (23.5%) in the PDA stent group, (*p* = 0.01), yet the need for inotropic support before the index procedure did not differ between the two groups. Two patients of the 92 patients in the BTTS group underwent attempted PDA stenting, unsuccessfully due to mal-positioning of the stent. Only ten (10.9%) of the BTTS group had undergone previous surgery or interventional procedure, including balloon atrial septostomy (*n* = 2), surgical pulmonary valvotomy (*n* = 4), central shunt (*n* = 1), pacemaker implantation (*n* = 1), and unsuccessful PDA stenting (*n* = 2). In contrast, 32.4% of the PDA stent patients (*n* = 11) had undergone previous intervention, including balloon atrial septostomy in one case, and balloon valvuloplasty with or without perforation of the pulmonary valve in the other ten cases.

**Table 1:**
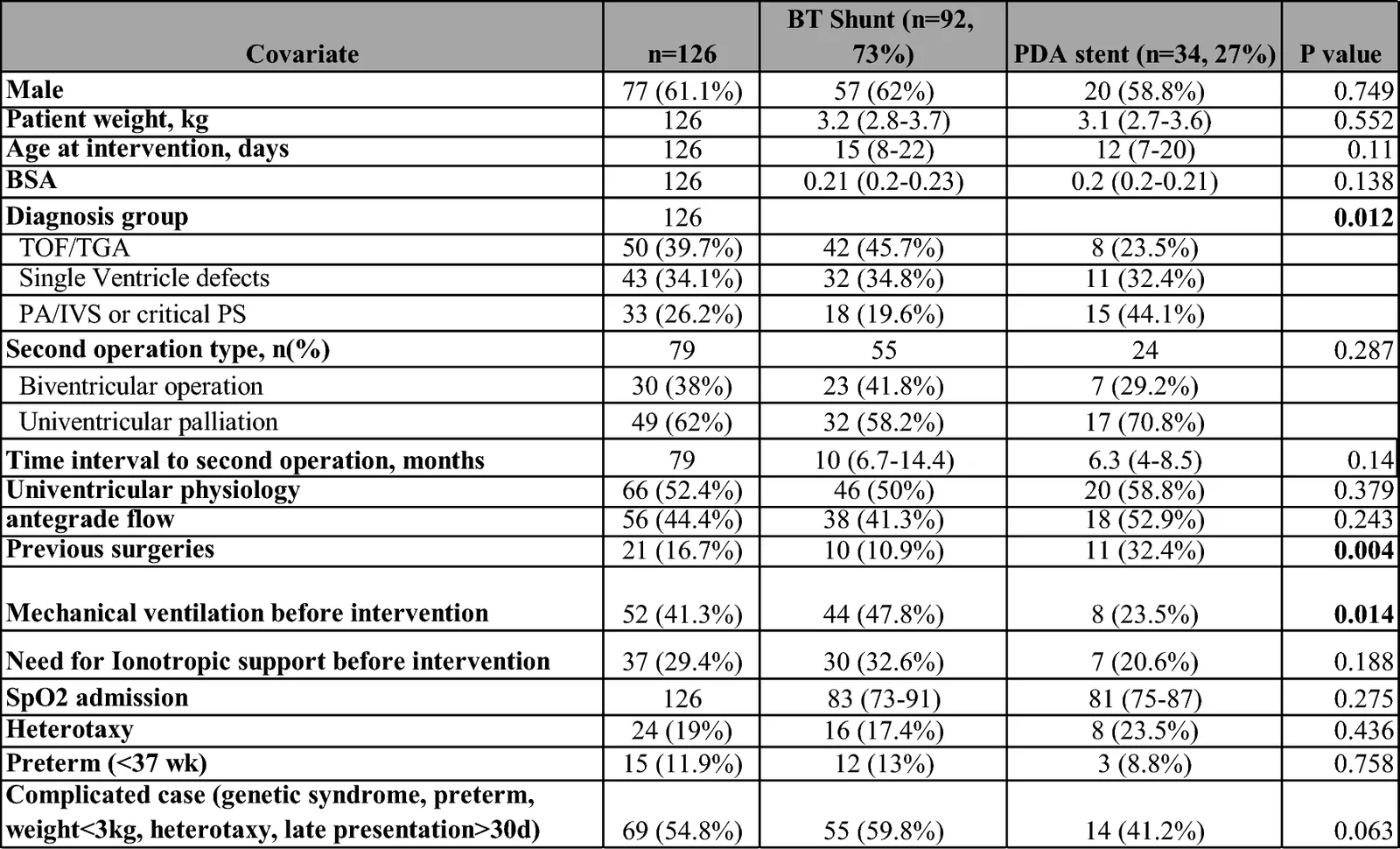
Patient demographics and clinical pre-intervention factors

Values are reported as *n* (%), median (25th–75th percentile) or mean (SD) when appropriate

*TOF* Tetralogy of Fallot, *TGA* Transposition of great arteries, *PA/IVS* pulmonary atresia, intact ventricular septum, *PS* pulmonary stenosis

This is due to the makeup of the PDA stent group, which included 15(44.1%) critical pulmonary stenosis or pulmonary atresia, intact ventricular septum (PA/IVS) patients, 53% of whom underwent univentricular palliation, as compared to 18(19.6%) in the BTTS group (*p* < 0.05), of whom 78% were deemed univentricular. On the other hand, the overall proportion of univentricular patients was not statistically different between the two groups (59% vs. 50%, respectively, *p* = 0.38), and these included babies with tricuspid atresia, double inlet left ventricle, Ebstein's anomaly, or complex single ventricle, all with duct dependent pulmonary circulation who underwent a Glenn anastomosis as their second stage operation. Babies with Tetralogy of Fallot, double outlet right ventricle, or transposition of the great arteries with severe pulmonary stenosis or atresia underwent subsequent biventricular repair including Tetralogy of Fallot repair, arterial switch operation, Rastelli procedure, or Double Switch procedure.

**Fig. 2:**
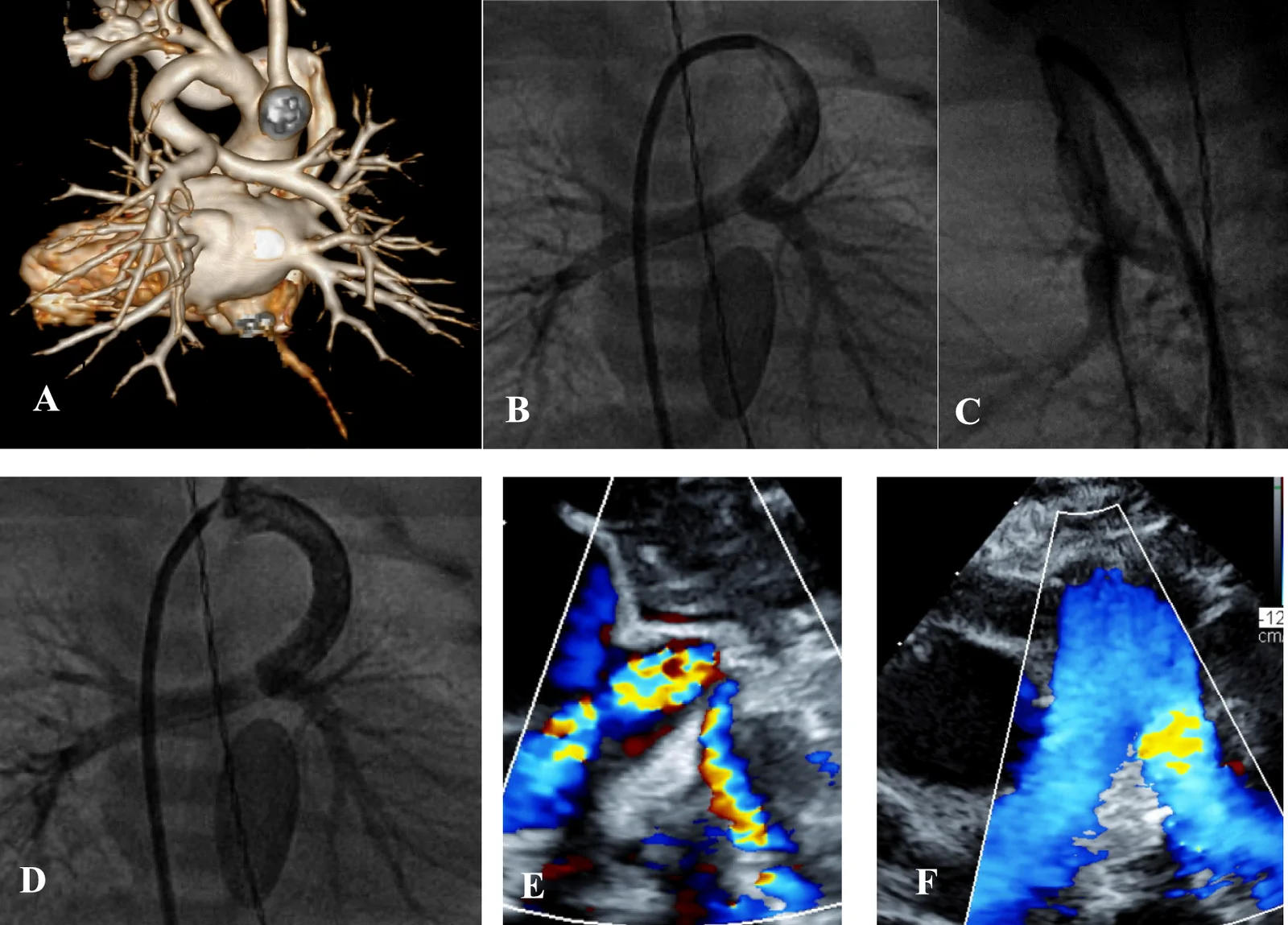
**A** 3D rendering of CT scan posterior view showing anatomy of a large PDA from the underside of the innominate artery and mild right pulmonary artery stenosis in a neonate with Tetralogy of Fallot, pulmonary atresia, and right aortic arch. **B** Angiography in anteroposterior view showing a large PDA supplying confluent branch pulmonary arteries and **C** Lateral view showing mild right pulmonary artery stenosis as seen on CT. **D** Angiography in anteroposterior and lateral views after PDA stenting with a 4*30 mm Onyx^TM^ Drug Eluting Stent, showing a well-seated stent and good flow to both pulmonary arteries. **E** Follow up echo six months after PDA stenting showing stenosis of the left pulmonary artery, and echo result **F** of no qualitative or quantitative pulmonary artery stenosis after Tetralogy of Fallot surgical repair with right ventricle to pulmonary artery homograft and pulmonary artery plasty

The two groups did differ in pulmonary artery diameter before intervention (Table 2). Qualitative PA stenosis was significantly more common in the PDA stent group 20 (58.8%) in comparison to the BTTS group 30 (33.7%), and echo measurements trended towards smaller PAs in the PDA stent group. Mean proximal RPA diameter on echocardiogram was 4.2 ± 1 mm, vs.4.6 ± 1.1 mm in the BTTS group, (*p* = 0.071) and mean proximal LPA diameter was 4.2 ± 1.2 mm, vs. 4.0 ± 1.0 mm, respectively (Fig. 2).

**Table 2:**
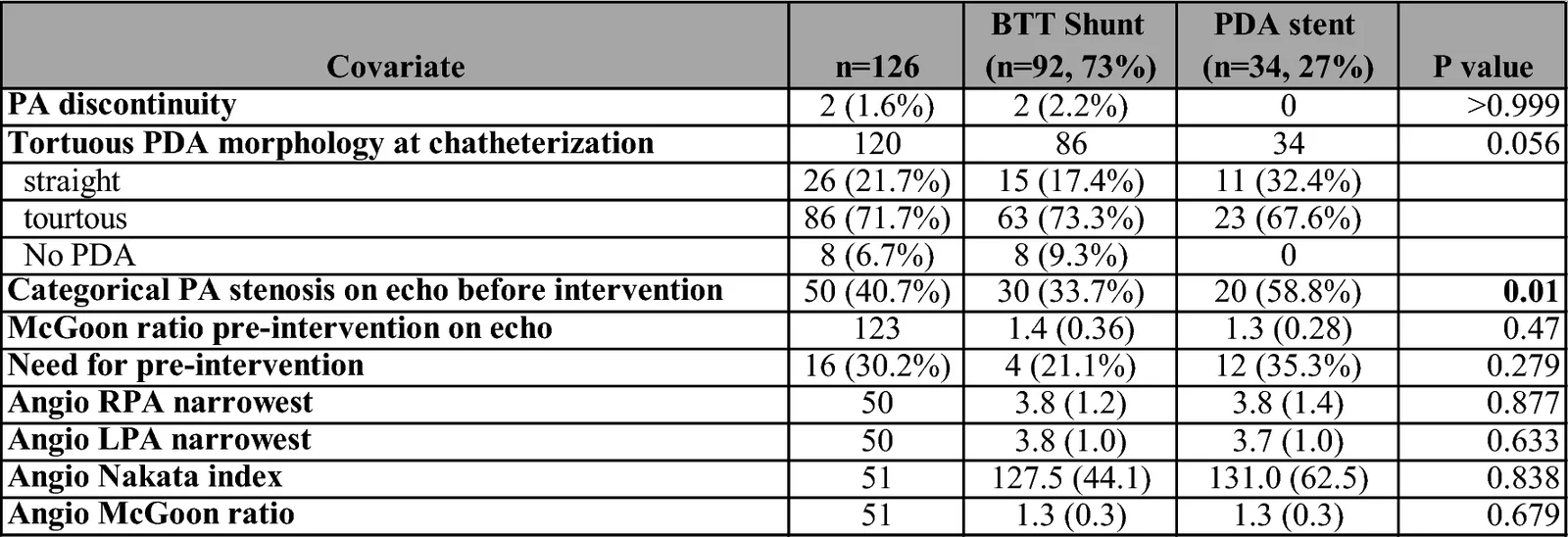
Pulmonary artery assessment before intervention

Values are reported as *n* (%) or mean (SD), when appropriate. The diameter of the pulmonary arteries is expressed in millimeters

*PA* pulmonary artery, *LPA* left pulmonary artery, *RPA* right pulmonary artery

### Post-intervention Outcomes

Post-procedural outcomes are summarized in Tables 3 and 4. In the PDA stent group, the duration of Intensive Care Unit (ICU) hospitalization and total hospitalization days after the intervention was significantly shorter. Mortality in the ICU 30 days after procedure was more common in the BTTS group, and the PDA stent group was ventilated for a significantly shorter period after the intervention. The need for ionotropic support as calculated by VIS at the day of intervention, one day and two days after intervention, was significantly higher in the BTTS group at all time-points except for seven days after intervention, when it was zero for all survivors in both groups. The BTTS group had significantly more complications compared to the PDA stent group, see Table 4, even though oxygen saturation (%SpO2) upon discharge was significantly higher in the PDA stent group (90 ± 5.2%) compared to BTTS group (85 ± 5.7%), (*p* < 0.001). The most common etiology for urgent catheterization or surgery after BTTS procedure was shunt thrombosis/stenosis or pulmonary artery stenosis. None of the children in the PDA stent group had stent or significant vascular access related complications. Any child with a weak femoral pulse after the procedure was treated with low molecular weight Heparin (subcutaneous LMWH), and there was no long-term arterial thrombosis.

**Table 3:**
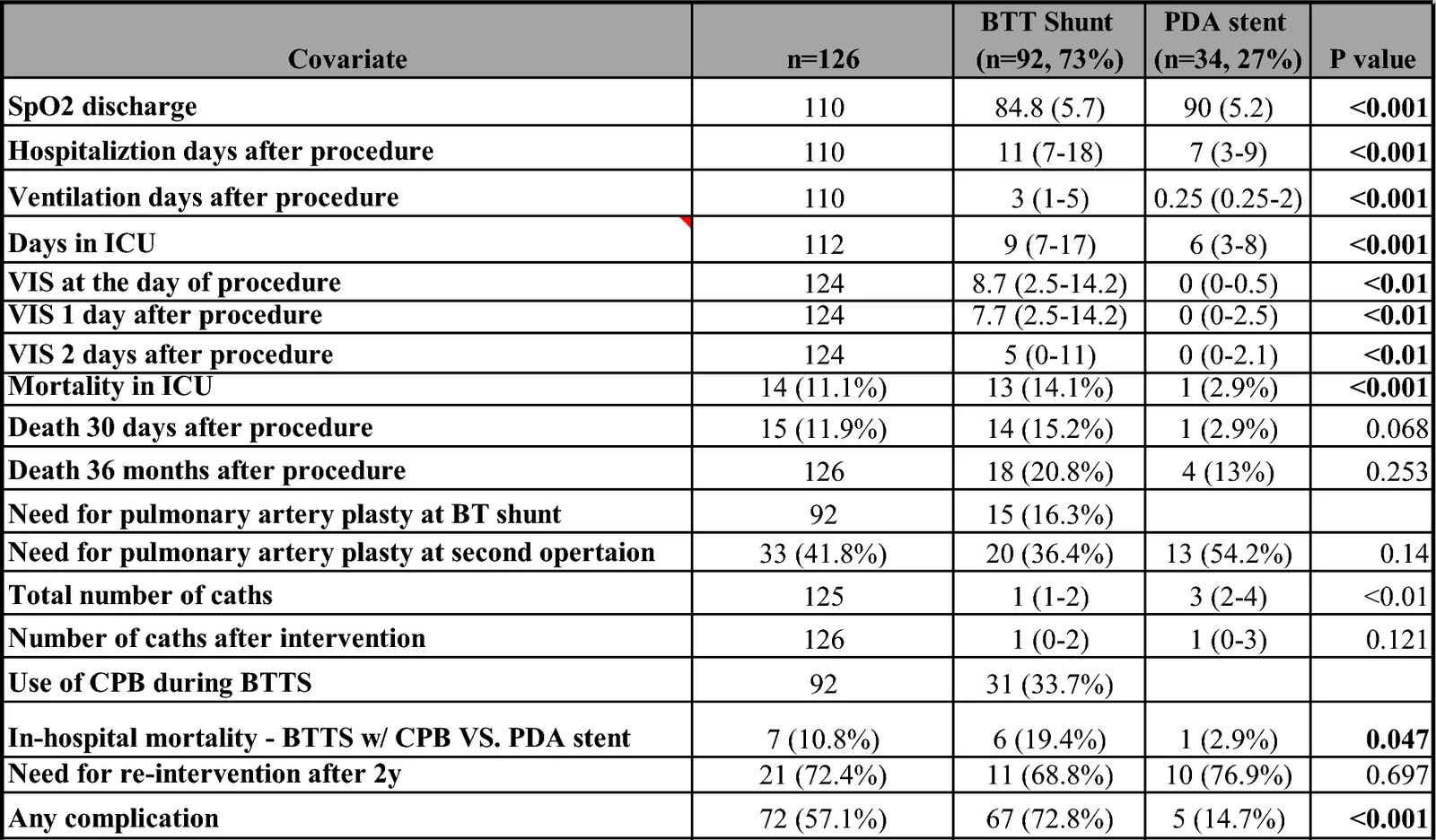
Post-intervention outcomes

Values are reported as *n* (%) or median (25th–75th percentile), when appropriate

*ICU* intensive care unit, *CBP* cardiopulmonary bypass, *VIS* Vasoactive Inotropic score [VIS = Dopamine dose (mcg/kg/min) + Dobutamine dose (mcg/kg/min) + 100 × epinephrine dose (mcg/kg/min) + 10 × Milrinone (mcg/kg/min) + 10,000 × Vasopressin dose (units/kg/min) + 100 × Norepinephrine dose (mcg/kg/min) [9]

**Table 4:**
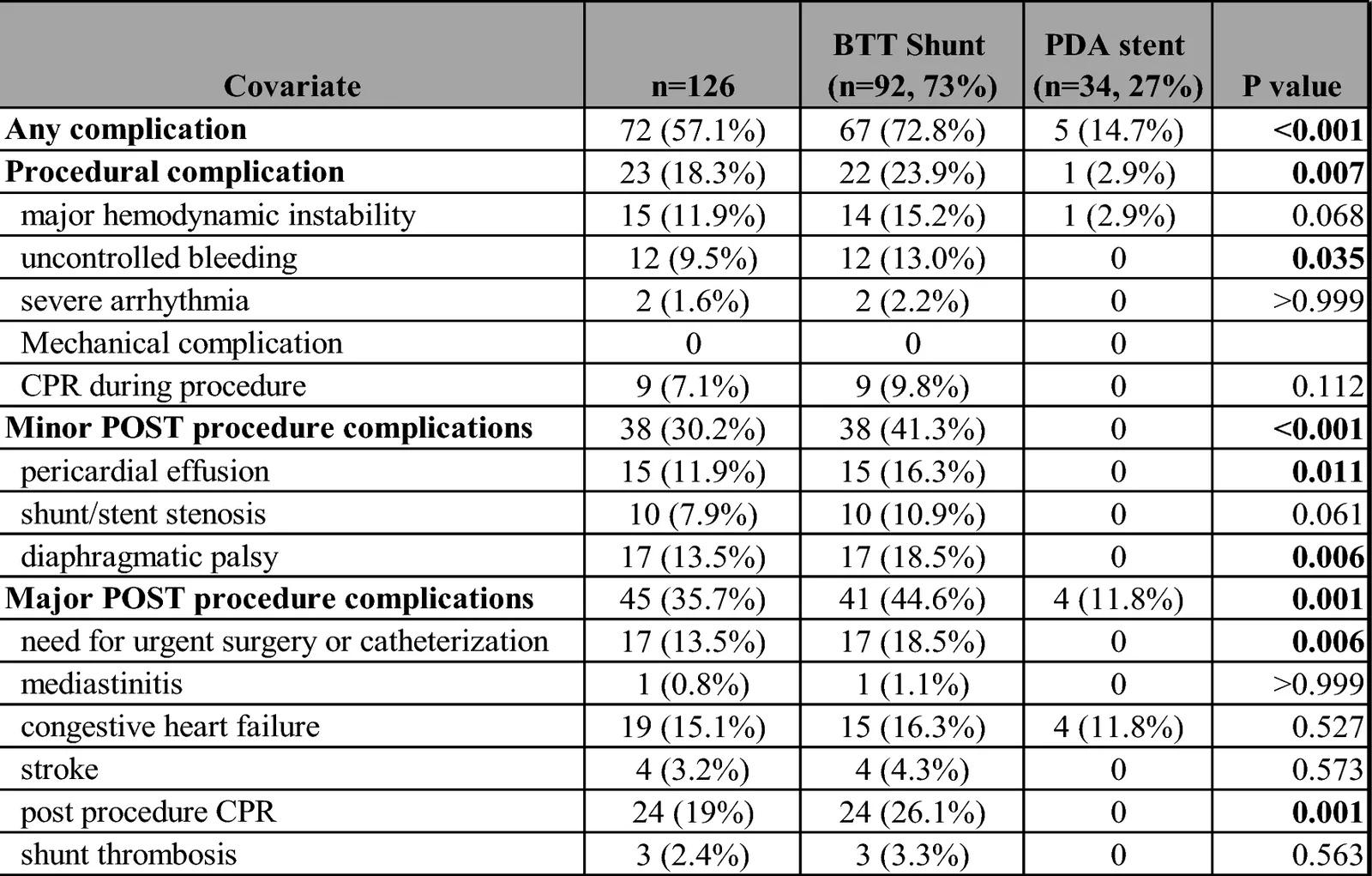
Complications

Values are reported as *n* (%) or median (25th–75th percentile), when appropriate

*CPR* cardiopulmonary resuscitation

A total of 31 (33.7%) patients who underwent BTTS under cardiopulmonary bypass (CBP) machine during the operation due to surgeon preference. Yet in-hospital mortality among this group of BTTS patients who underwent CPB was significantly higher 6(19.8%) than that in the PDA stent group 1(2.9%), (*p* = 0.047). Mortality in the ICU was also significantly higher in the entire BTTS group, 14.1% vs. 2.9% of the PDA stent group (*p* < 0.001), and mortality 30 days after intervention, although not statistically significantly different, trended towards being higher in the shunt patients (15.2% vs. 2.9%, *p* = 0.068).

**Fig. 3:**
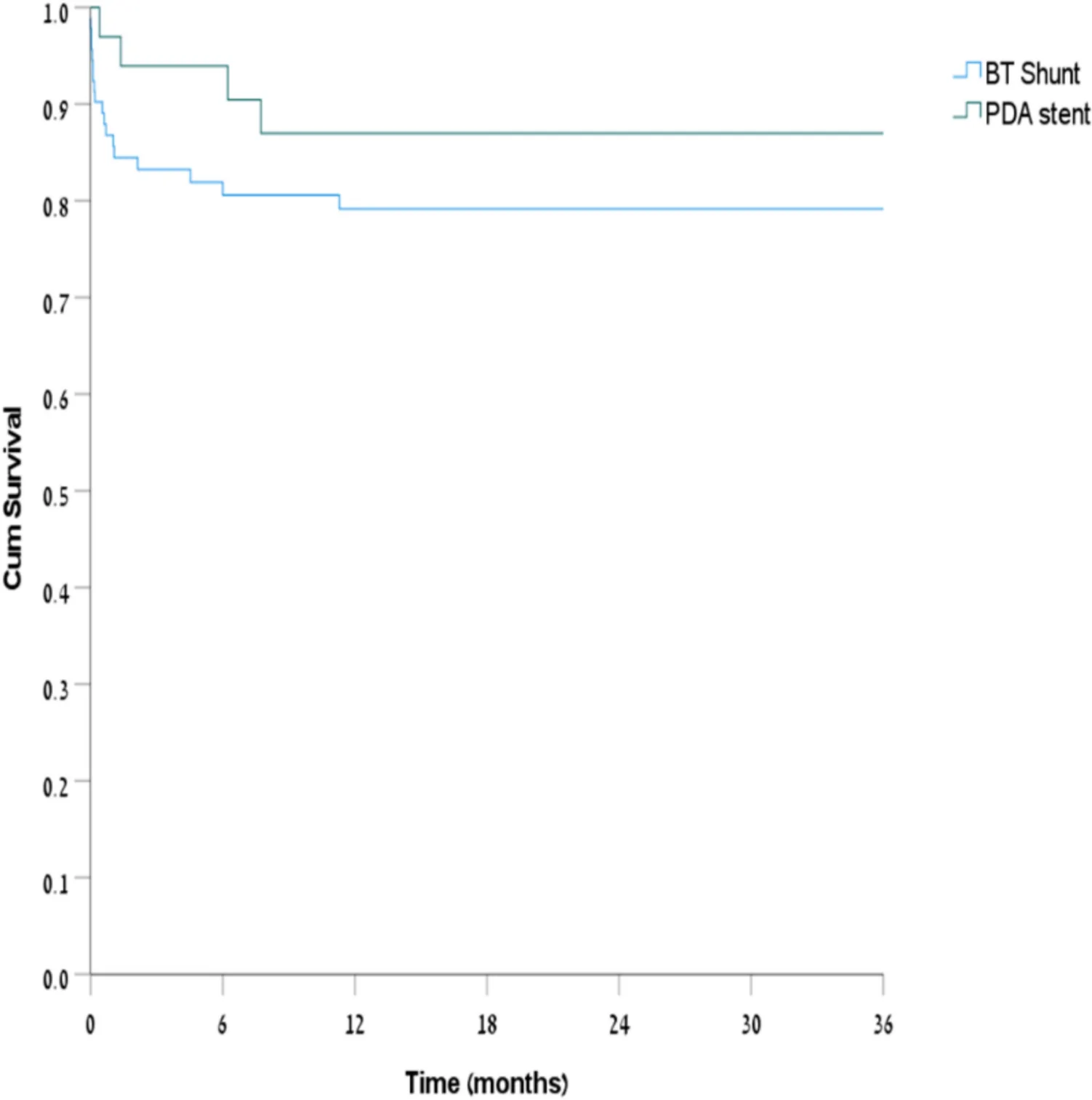
Kaplan–Meier Curve comparing survival in patients with PDA stenting and those with BTTS (*p* = 0.253)

There was no difference between the two groups in the percent of patients who required reintervention after six months: 9 in the BTTS group (23.7%) vs. 6 in the PDA stent group (18%). Reintervention included pulmonary valve, pulmonary artery, or shunt balloon dilation, pulmonary artery or shunt stent, collateral or shunt embolization, and balloon atrial septostomy. Interestingly, overall survival 36 months after intervention, as demonstrated by the Kaplan–Meier curve in Fig. 3, was not statistically different between the two groups, 79.2% in the BTTS group vs. 87% survival in the PDA stent group, (*p* = 0.253), yet this is likely not related to the initial palliation procedure.

## Categorical and Quantitative PA Assessment on Echocardiogram and Angiography Six Months and Two Years After Intervention

**Fig. 4:**
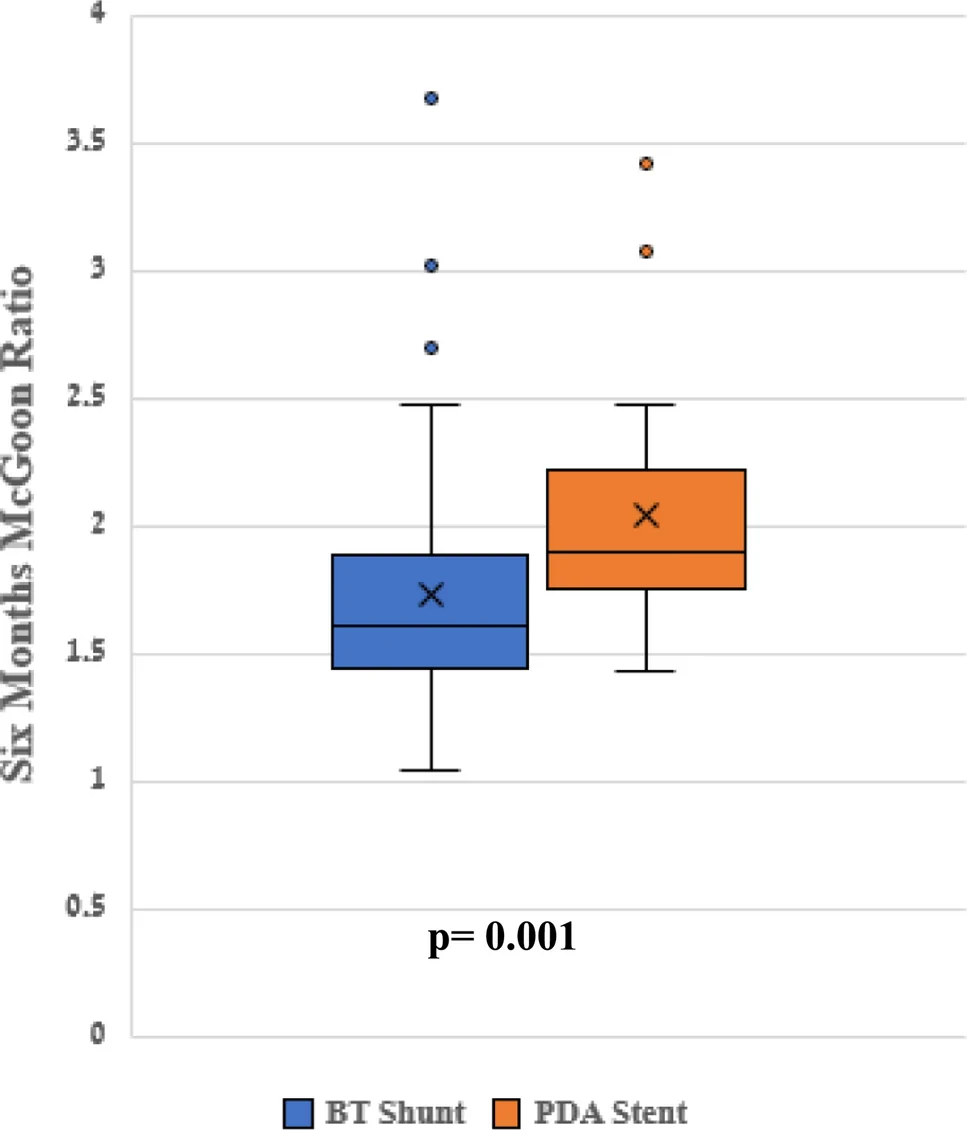
Box-and-whisker plot demonstrating the difference in pulmonary artery size as measured by McGoon ratio six months after intervention

Categorical and quantitative PA assessment on echocardiogram and angiography over the course of 6 months and 2 years after initial intervention, are summarized in Tables 5 and 6. Echocardiograms 6 months after intervention demonstrated higher McGoon ratio among the PDA stent group 1.9 mm (1.7–2.2) compared to BTTS 1.7 mm (1.4–1.9), (*p* = 0.001) (Fig. 4). In addition, the BTTS group had significantly more RPA stenosis on angiography 6 months after intervention, and 12 (37.5%) of the BTTS group had shunt stenosis while no significant stent stenosis was observed in the PDA stent group (*p* = 0.004).

**Table 5:**
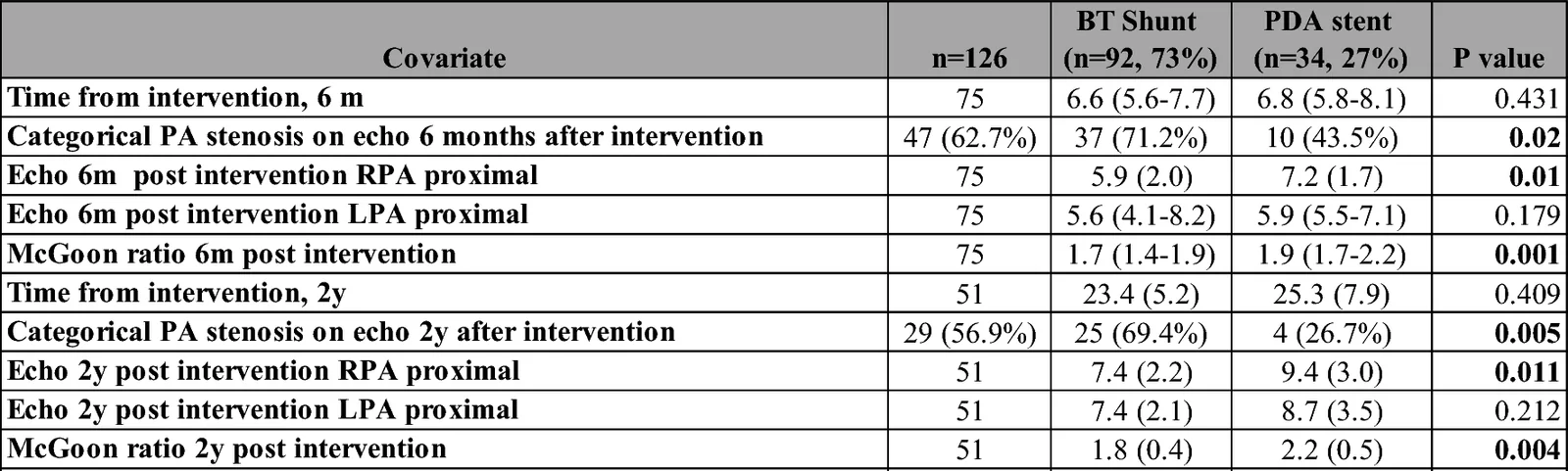
Categorical and quantitative PA assessment on echocardiogram 6 months and 2 years after intervention

Values are reported as *n* (%), median (25th–75th percentile) or mean (SD) when appropriate. The diameter of the pulmonary arteries is expressed in millimeters

**Table 6:**
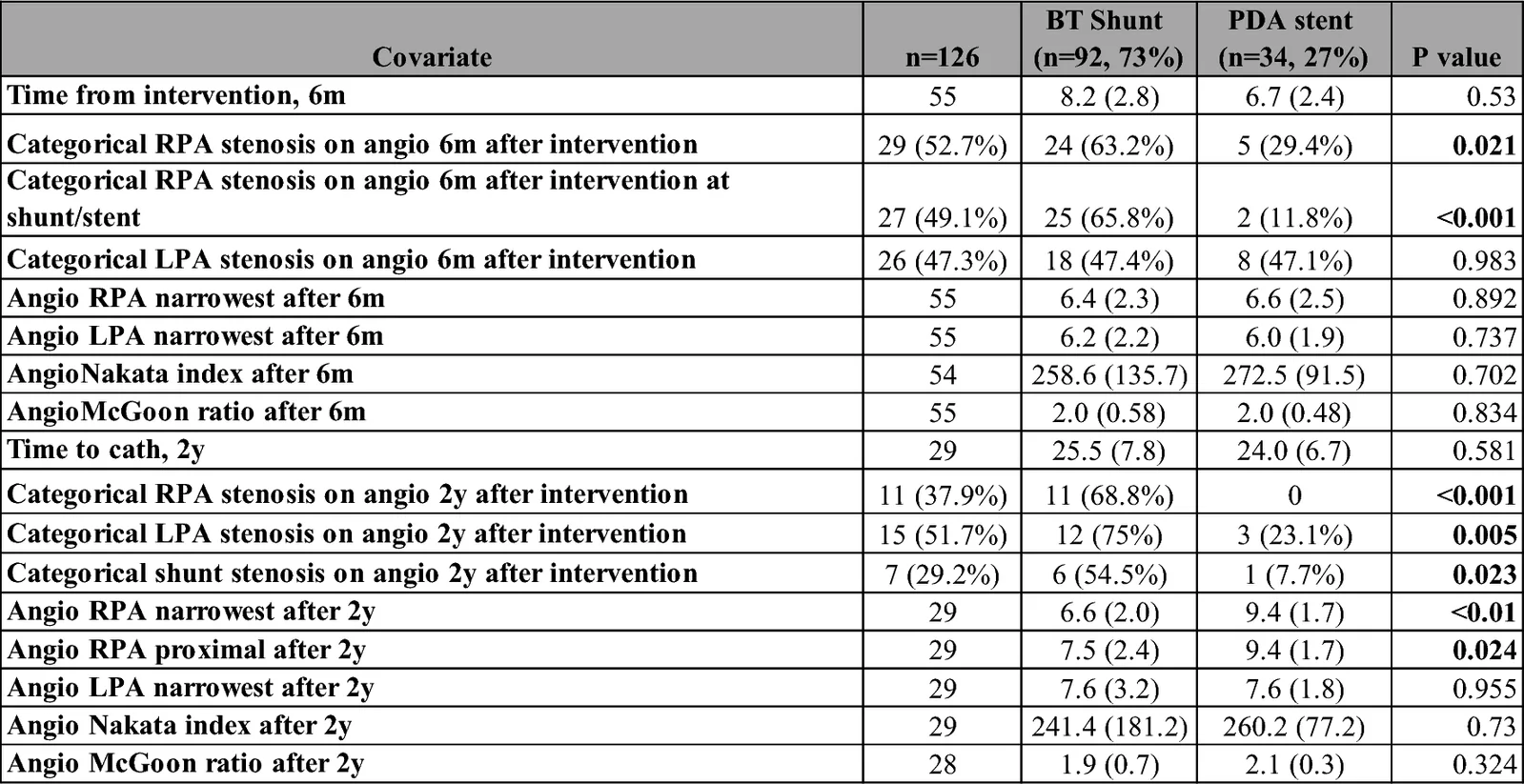
Categorical and quantitative PA assessment on angiography—6 months and two years after intervention

Values are reported as *n* (%), median (25th–75th percentile) or mean (SD) when appropriate. The diameter of the pulmonary arteries is expressed in millimeters

**Fig. 5:**
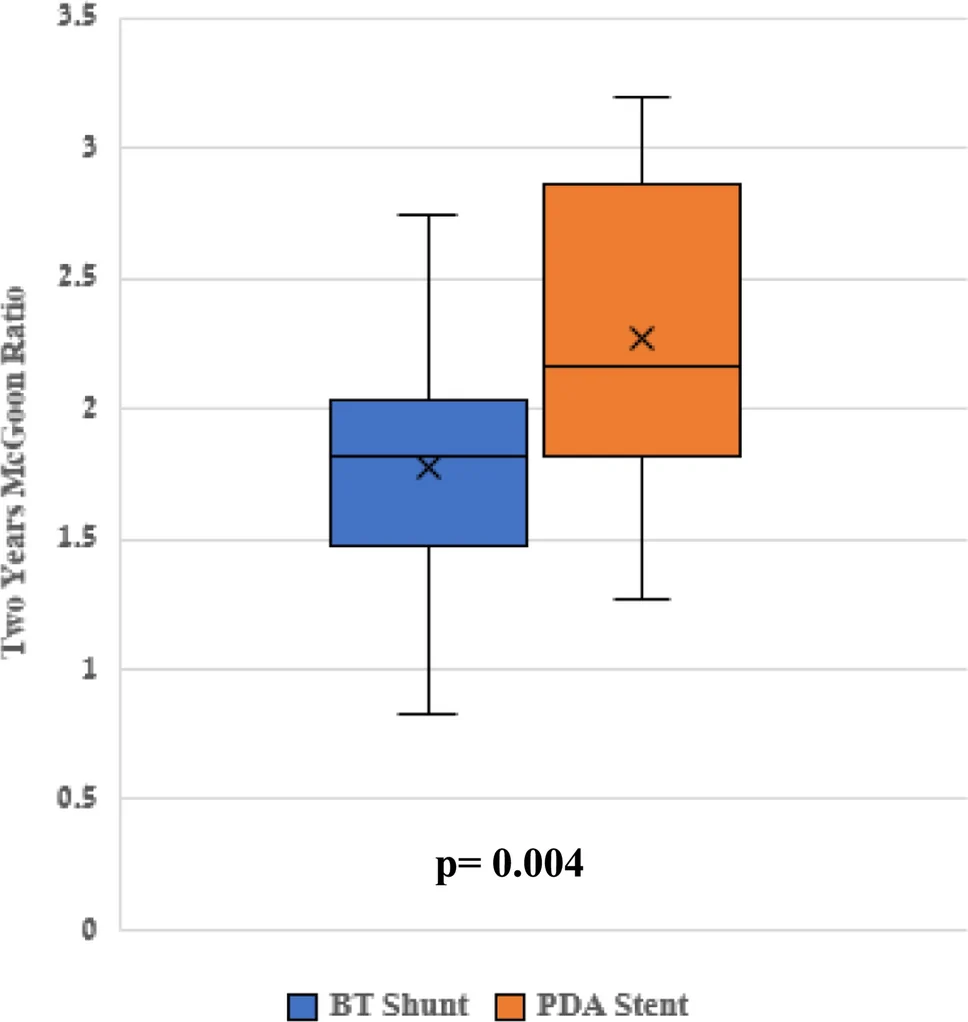
Box-and-whisker plot demonstrating the difference in pulmonary artery size as measured by McGoon ratio two years after intervention

Echocardiograms 2 years after intervention also demonstrated significant differences between the groups. 25 (69.4%) of the BTTS group had PA stenosis, while only 4 (26.7%) in the PDA stent group did (*p* = 0.005). McGoon ratio was higher in PDA stent group with a mean of 2.2 ± 0.5 compared to the BTTS group 1.8 ± 0.4, (*p* = 0.004) (Figure 5). Angiographically, 11 (68.8%) of the BTTS group had RPA stenosis and 12 (75%) had LPA stenosis, while no RPA stenosis was observed in the PDA stent group and LPA stenosis was present in only in three babies six months after the procedure (23.1%) (*p* < 0.001, *p* = 0.005, respectively). What is more, two years after the initial procedure mean RPA diameter was 9.4 ± 1.7 mm in the PDA stent group vs. only 7.5 ± 2.4 mm in the BTTS group (*p* = 0.024).

## Comparison of Pulmonary Artery Stenosis Between Patients Who Underwent Arterial Plasty During the First or Second Intervention

**Fig. 6:**
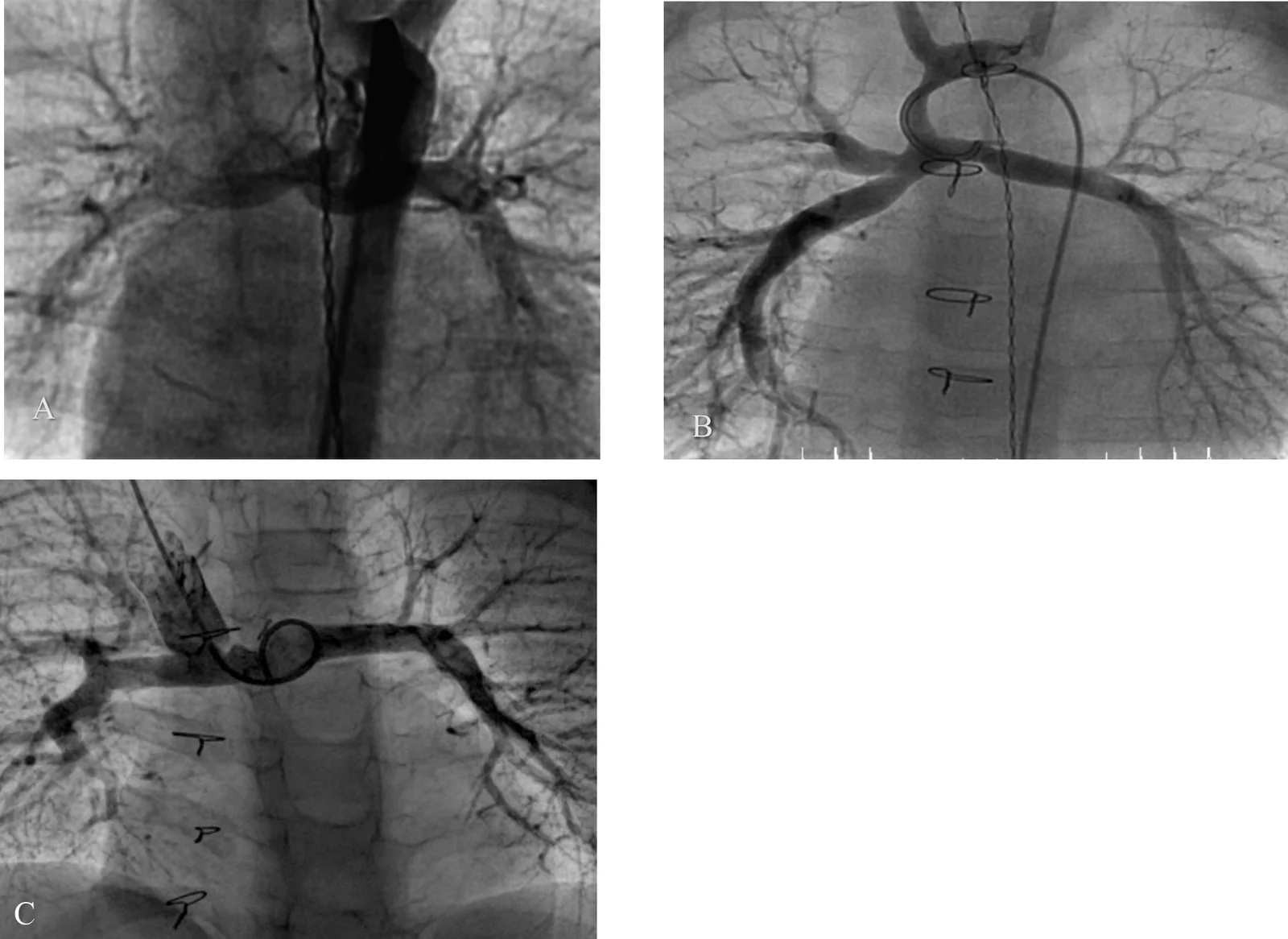
Patient 1: **A** Neonate referred for surgical BTTS & pulmonary artery plasty due to branch pulmonary artery stenosis, RPA = 3 mm (z score − 3.7), LPA 3.7 mm (z score − 1.9) at hilum, Nakata index = 89 mm^2^/m^2^, McGoon's ratio = 1.03 **B** Bilateral pulmonary stenosis at pre-Glenn cath following neonatal pulmonary artery plasty, RPA = 4.6 mm (z score − 2.7), LPA 3.9 mm (z score − 3.7) at hilum, Nakata index = 82 mm^2^/m^2^, McGoon's ratio = 1.12 **C** Pre-Fontan cath showing poor pulmonary artery growth bilaterally, RPA = 5.5 mm (z score − 3), LPA 5.2 mm (z score − 3.2) at hilum, Nakata index = 83 mm^2^/m^2^, McGoon's ratio = 1.3

**Fig. 7:**
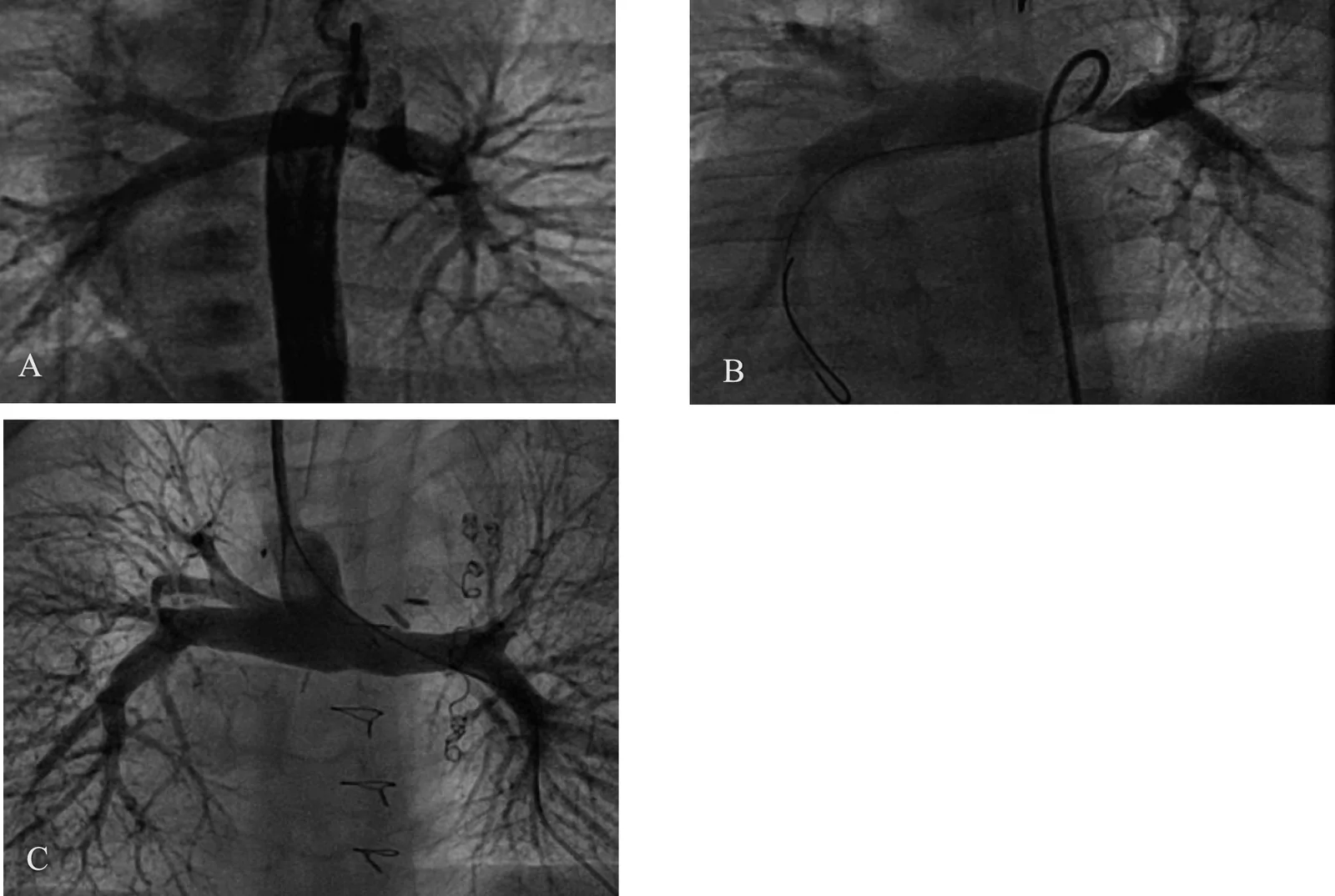
Patient 2: **A** Neonate with branch pulmonary artery stenosis, imaged prior to ductal stenting, RPA = 4.2 mm (z score − 1.2), LPA 4 mm (z score − 1.3) at hilum, Nakata index = 132 mm^2^/m^2^, McGoon's ratio = 1.28 **B** Left pulmonary artery stenosis at stent insertion seen on pre-Glenn cath following neonatal PDA stenting, RPA = 11.2 mm (z score + 4.6), LPA 7.9 mm (z score + 2.1) at hilum, Nakata index = 492 mm^2^/m^2^, McGoon's ratio = 1.79 **C** Pre-Fontan cath showing good result of pulmonary arterioplasty performed at the Glenn operation, RPA = 10.2 mm (z score + 1.2), LPA 7.3 mm (z score − 1.1) at hilum, Nakata index = 206 mm^2^/m^2^, McGoon's ratio = 1.88

Three patients underwent surgical BTTS and PA plasty at first intervention, 4 patients underwent BTTS as first intervention and PA plasty at second operation and 6 patients from the PDA stent group underwent PA plasty at the second operation. Qualitative PA stenosis was assessed 2 years after initial intervention, and comparison between these three groups was made (Table 7). At two-year assessment, RPA stenosis was present in all three (100%) of the patients who had undergone BTTS and pulmonary artery repair at the first operation and in three (75%) of the patients who had PA plasty at their second operation following an initial BTTS. In contrast, no stenosis was present in patients who underwent PDA stent as first intervention and correction of the pulmonary artery at the following operation (*p* = 0.005). Stenosis at the shunt site was present in 3 (100%) of the patients who underwent BTTS and PA plasty at the first operation and in 2 (50%) of the patients underwent BTTS as first intervention and PA plasty at their second operation, while there was no stent stenosis among the PDA stent group who underwent PA plasty at the second operation, (*p* = 0.016). Figures 6 and 7 demonstrate examples of the imaging of a patient who underwent BTTS with attempted early PA plasty vs. the pulmonary artery growth in a patient who underwent initial PDA stent with subsequent PA plasty.

**Table 7:**
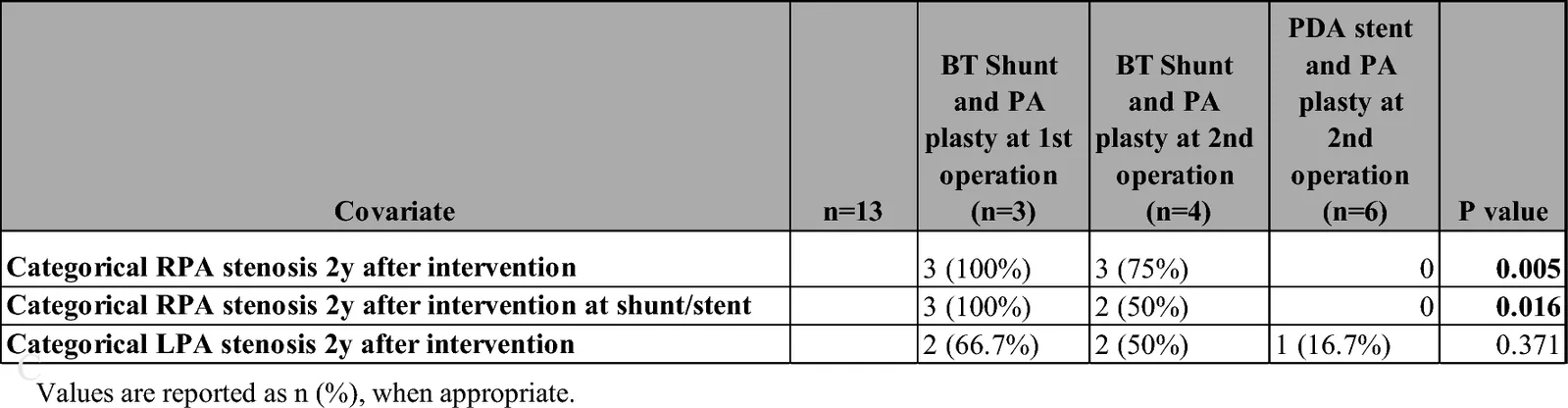
Comparison of PA stenosis between patients who underwent pulmonary artery plasty during the first or second intervention

Values are reported as *n* (%), when appropriate

## Discussion

In this study, we aimed to assess the morbidity and mortality associated with BTTS, in comparison to PDA stenting, as well as the effect on pulmonary artery growth over the course of time. Most of the patient demographics and preoperative conditions did not differ. There were significantly more patients diagnosed with pulmonary atresia/intact ventricular septum or critical pulmonary stenosis in the PDA stent group, probably due to center preference, as seen in other studies such as Glantz et al. and McMullan et al. [7, 10]. Although the PDA morphology in patients with pulmonary atresia tends to be more favorable, making the ductal stenting technically simpler [11], the two overall groups did not differ in terms of PDA tortuosity. Furthermore, there was no difference between the numbers of patients with univentricular physiology or antegrade flow in the two groups, so we do not believe that the percent of pulmonary atresia/stenosis patients accounted for the overall improved outcomes of the PDA stent group.

The need for mechanical ventilation before the procedure was significantly more common in the BTTS group. Many of the patients who were ventilated before the procedure were transferred from other medical centers due to a lack of appropriate diagnostic and treatment equipment. It was decided not to extubate these patients before the procedure. In contrast, those born at our medical center often had a prenatal diagnosis of the specific cardiac defect and were monitored and treated with Prostaglandin E, eliminating the need for mechanical ventilation. If the initial ventilation status were the only factor influencing outcomes, we would expect post-procedure ventilation days to be roughly twice as high in the BTTS group. However, we observed that post-procedure ventilation days were actually ten times higher in the BTTS group compared to the PDA stent group.

The prevalence of previous surgeries before the initial palliation was more common among the PDA stent group. The patients in PDA stent group who underwent previous procedures were mostly patients diagnosed with pulmonary atresia or critical pulmonary stenosis. At our center, these patients are initially treated with pulmonary valve perforation and dilation percutaneously, then a wait-and-see period to assess if they can be weaned from oxygen support and prostaglandin E treatment. If not, they are taken to another catheterization for PDA stenting.

When we first performed PDA stenting our center, only babies with high risk for surgery and two sources of pulmonary blood flow were referred for this procedure. Over the years of the study, PDA stenting has become the go-to intervention for babies with duct dependent pulmonary circulation. Therefore, the surgical vs. stenting groups assessed did not differ in terms of antegrade blood flow or complicated pre-interventional findings, and we believe that this era difference did not have significant effect. There was no significant difference in terms of pulmonary artery measurements prior to intervention. If anything, the ductal stenting group exhibited qualitatively more pre-intervention pulmonary artery stenosis.

Our study demonstrates that PDA stenting was associated with significantly shorter ICU length of stay, shorter hospitalization, fewer ventilation days, and less need for inotropic support after the procedure. A large meta-analysis carried by Alsagheir et al. exhibited similar results with a median of 4.6 days in ICU after PDA stent and 5.8 days of hospital length of stay [8], and a similar trend was demonstrated by Ratnayaka et al. [12]. Bentham et al. also showed the length of ventilation was significantly shorter after PDA stenting with a median of 1 day after the procedure, compared to 4 after BTTS. Longer hospitalizations as well as the use of mechanical ventilation increase the risk for serious hospital-acquired infections and other complications [13]. Findings of less need for inotropic support are also of great importance, reflecting the improved hemodynamic condition of the PDA stent patients after intervention. A high VIS score, associated with morbidity, mortality, and poor clinical outcome in infants undergoing cardiac surgery [9], is reflected in our findings that the BTTS group had higher ICU mortality.

All patients in the BTTS group received a 4 mm shunt, regardless of age and weight, with a median weight of 3.2 kg in the study group. This uniform shunt size may have contributed to post-procedure over-circulation and related complications, such as major hemodynamic instability and congestive heart failure, which were observed more frequently in the BTTS group. In contrast, PDA stenting conforms to the patient’s anatomy, resulting in fewer over-circulation complications, and stent diameter was adjusted according to patient weight: 2.5 kg babies received a 3.5 mm diameter stent, whereas babies above 3 kg received a 4 mm stent.

The high cardiopulmonary bypass rate in patients who underwent BTTS, was primarily due to surgical preference aimed at improving the anastomosis site of the BTTS, rather than concomitant surgery. However, as demonstrated in our study, the use of cardiopulmonary bypass did not improve surgical outcomes and instead led to higher morbidity and mortality, suggesting that ductal stenting, a less invasive procedure has significant survival advantages. Bentham et al. concluded that since PDA stenting eliminates the need for thoracic surgical dissection of the neonate, it may be related with better post-procedural stability and survival [14]. What is more, babies with branch pulmonary artery stenosis referred for initial shunt with PA plasty, actually demonstrated more pulmonary artery stenosis during follow-up. In comparison, babies with neonatal branch PA stenosis who had initial ductal stenting followed by pulmonary artery repair at the second stage procedure, exhibited significantly better pulmonary artery growth. Although the number of patients compared in this respect are small, the results are very suggestive, and may be due to the improved ease of performing surgical arterial repair in larger babies (Figs. 4, 5) [15].

**Fig. 8:**
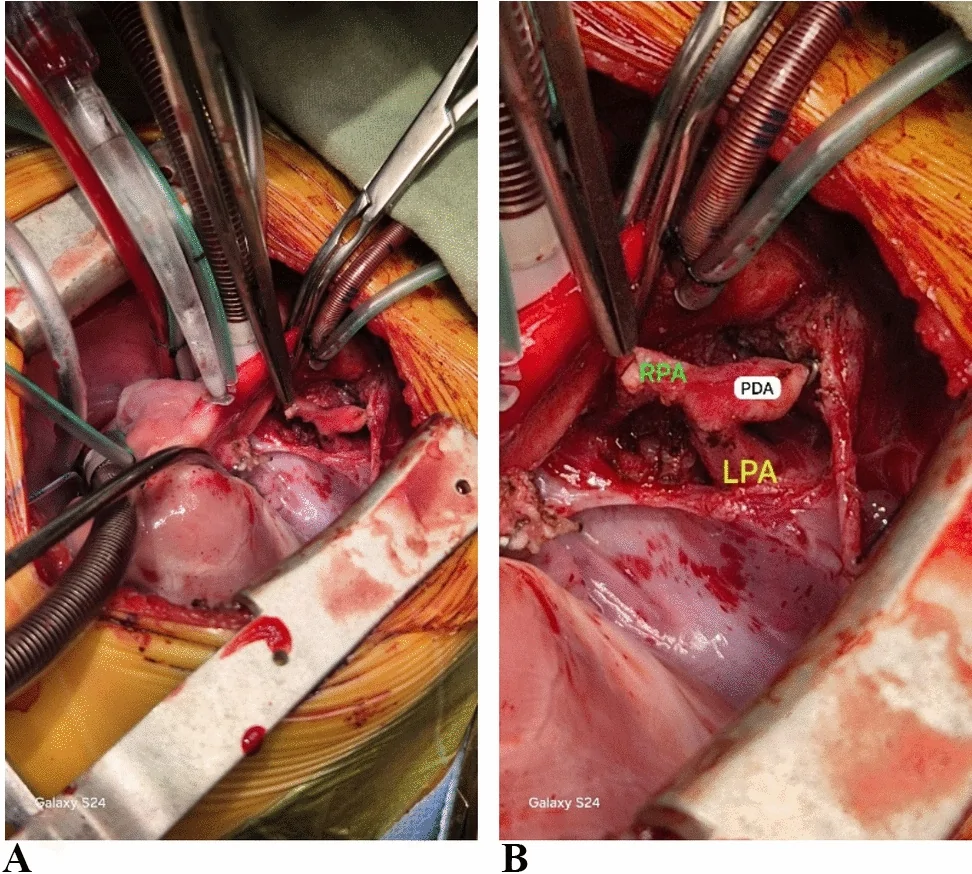
Surgical images showing **A** the PDA stent in situ as encountered by the surgical team at the definitive operation nine months after implantation, and **B** following removal of the stent, prior to arterioplasty. Surgical video available in Online Appendix

Li et al. assumed that preforming BTTS with concomitant pulmonary arterioplasty may have favorable results on pulmonary blood flow. However, it was discovered that when these two procedures were combined, there was a higher rate of hospital mortality [16]. In our study we examined the mid-term effects of combining two procedures, BTTS and PA plasty as initial surgery, on mid-term PA stenosis, in comparison to patients who underwent BTTS or PDA stenting at first, then pulmonary artery repair during their second surgery. Our findings indicate that patients who underwent BTTS along with PA plasty as part of an initial surgical procedure had a higher rate of RPA stenosis and shunt site stenosis compared to those who underwent PDA stenting followed by pulmonary arterioplasty at the second procedure. It therefore appears that a child with duct dependent pulmonary blood flow presenting with pulmonary artery stenosis at birth is better palliated with an initial PDA stent until pulmonary artery plasty is safely performed surgically on larger, more developed pulmonary arteries later in infancy (Fig. 8).

Santoro et al. found that both BTTS and PDA stent groups experienced significant PA growth as determined by the Nakata index and McGoon ratio, with no difference in the overall growth of the pulmonary arteries. However, the group treated with PDA stents had more symmetrical growth of the pulmonary arteries as measured by the ratio of left pulmonary artery to right pulmonary artery diameter [17]. We also found that the BTTS group experienced a significantly narrower proximal RPA diameter over time, which may have been caused by the shunt implantation distorting the PA anatomy. This suggests that the BTTS group may have less favorable mid-term effects such as PA distortion and stenosis at the shunt site [11]. In addition, although the PDA stent group had a higher prevalence of pulmonary artery stenosis before initial intervention, they experienced significantly better PA growth compared to the BTTS group as expressed in a higher McGoon ratio.

Given the small size of the pulmonary arteries in these infants, the small study group, and the difficulty achieving statistically significant quantitative differences, we decided to include a qualitative assessment of the pulmonary arteries. This assessment was defined as a 50% reduction in PA diameter. We believe the categorical assessment holds clinical importance since clinical decisions are often based on qualitative evaluations rather than specific numerical measurements.

## Conclusions

Our study provides insights into the morbidity and mortality associated with BTTS compared to PDA stenting, along with their effects on pulmonary artery growth. PDA stenting demonstrated shorter ICU and hospital stays, lower dependency on ionotropic support, and fewer procedural complications compared to BTTS, indicating its superiority in terms of safety and clinical outcomes. Additionally, PDA stenting offers symmetrical and potentially better pulmonary artery growth. Moreover, performing BTTS on cardiopulmonary bypass, while initially thought to benefit pulmonary blood flow, actually led to higher hospital mortality. Our study suggests that PDA stenting may be a better option especially for infants with pulmonary artery stenosis, recommending initial palliation with a PDA stent followed by pulmonary arterioplasty later in infancy.

## Limitations

Our study had several limitations. First, it included only 126 patients, Secondly, the study is retrospective and nonrandomized, which could have introduced bias in our evaluation of the effectiveness of the palliative options. In addition, the number of patients with preoperative angiograms was relatively small, since most of the BTTS group did not undergo angiographic pulmonary artery assessment. The use of both echo and angiographic measures of pulmonary artery assessment, and of as many clear-cut clinical outcome measures as possible, was used to mitigate this. Many of the patients in our study were not residents of Israel, which made it difficult for them to come in for routine follow-ups in our medical center. As a result, their current status may be unknown, and we had limited imaging data to work with in order to evaluate the mid-term effects of each intervention. We therefore used a Kaplan–Meier survival curve based on each patient's last-known status, as well as set time-points for imaging assessment, to standardize findings as much as possible. Lastly, our sample size was small, specifically when comparing PA plasty during first and second intervention to allow meaningful comparison. We therefore recommend that this subject be studied in other medical centers that perform stenting across branch PA stenosis. At our center, this procedure was not commonly performed due to surgical preferences.

## Supplementary Information

Below is the link to the electronic supplementary material.

Supplementary file1 (MP4 29373 kb)

## Data Availability

No datasets were generated or analysed during the current study.
